# Associations between Accommodative Facility, Age, and Refractive Errors in Early, Older Adolescent Myopes and Emmetropes

**DOI:** 10.22599/bioj.284

**Published:** 2023-03-31

**Authors:** Dharani Ramamurthy, Hema Radhakrishnan, Shahina Pardhan

**Affiliations:** 1 SRM Medical College Hospital & Research Centre, SRM Institute of Science and Technology, IN; 2 Faculty of Biology, Medicine and Health, The University of Manchester, GB; 3 Vision and Eye Research Institute, School of Medicine, Anglia Ruskin University, GB

**Keywords:** Near accommodative facility, myopia, adolescents, age, refractive error

## Abstract

**Background:**

Accommodative functions are known to differ between myopes and emmetropes. It is not known whether accommodative facility differs at near between younger adolescent and older adolescent myopes and emmetropes.

**Aim:**

To examine whether accommodative facility differs at near between younger and older adolescent myopes and emmetropes.

**Methods:**

119 participants aged between 11–21 years were recruited. Refractive error was measured using cycloplegic retinoscopy. Near monocular accommodative facility was measured for 60 seconds, using a +2.00D/–2.00D handheld flipper and N6 print at 40 cm. Participants were classified into two age groups: (i) younger adolescents (range: 11–14 years) and (ii) older adolescents (range: 15–21 years). The criterion applied to define myopia was spherical equivalent refraction: ≥–0.50D) and spherical equivalent refraction: –0.25D to +0.75D) for emmetropia. Univariate Analysis of Variance was carried out to analyze the interaction of age groups and refractive groups on near accommodative facility.

**Results:**

Near monocular accommodative facility was significantly lower (p = 0.003) in younger adolescents (5.87 ± 3.72 cpm) compared to older adolescents (8.11 ± 4.11 cpm), indicating age as a significant main effect (F_1,115_ = 13.44; *p* = 0.0001). Younger adolescent emmetropes (4.77 ± 2.05 cpm, p = 0.005) and younger adolescent myopes (6.48 ± 4.12 cpm, p = 0.022) had significantly lower monocular near accommodative facility compared to older adolescent emmetropes (9.52 ± 3.27 cpm), but did not show any difference when compared to older adolescent myopes (p > 0.05). This indicates a significant association linking age and refractive error to near accommodative facility (F_1,115_ = 4.60; *p* = 0.03).

**Conclusion:**

Younger adolescent myopes and younger adolescent emmetropes had reduced monocular near accommodative facility than older adolescent emmetropes, but not when compared to older adolescent myopes.

## Introduction

Refractive error is known to influence various accommodative functions including accommodative response, accommodative convergence to accommodation (AC/A ratio), dark focus of accommodation or tonic accommodation, nearwork-induced transient myopia (NITM), and accommodative facility and response times (Logan et al. 2021).

Accommodative facility is an important visual function to measure, especially for children and adolescents whose visual tasks involve more frequent changes in fixation from near to a distance and vice versa. Positive response times are measured as the time taken to accommodate in response to the minus lens, and negative response time is measured as the time taken to relax accommodation in response to the positive lens (Pandian et al. 2006). Response times are measured using semiautomated flippers — which use a handheld flipper and computer software that automatically calculates response times (Pandian et al. 2006). Accommodative facility is measured as cycles per minute (cpm), with a delayed response time indicating reduced accommodative facility, while a faster response time indicates increased accommodative facility. Earlier research studies on accommodative facility have reported significant differences between myopic and emmetropic participants for distance, but not for near accommodative facility. O’Leary and Allen (2001) measured and compared monocular distance and near accommodative facilities using handheld flippers and reported a significant difference in distance accommodative facility between 18 to 27-year-old myopes (9.7 ± 6.3 cpm) and emmetropes (15.6 ± 6.8 cpm), but not for near accommodative facility (myopes: 11.4 ± 5.1 cpm; emmetropes: 12.9 ± 6.4 cpm). Radhakrishnan, Allen and Charman (2007) showed similar results among 20 to 35-year-old young adults for monocular distance and near accommodative facilities, measured using semiautomated flippers and power refractors, with myopes having significantly reduced distance accommodative facility (13.9 ± 2.2 cpm) compared to emmetropes (17.9 ± 3.9 cpm); however, monocular near accommodative facility was not significantly different between myopes (10.5 ± 1.6 cpm) and emmetropes (13.2 ± 1.6 cpm). These results were consistent among 6 to 8-year-old children for monocular distance and near accommodative facility testing using semiautomated flippers, with myopic children having significantly reduced accommodative facility for distance (5.5 ± 2 cpm) compared to emmetropic children (6.9 ± 1.7 cpm), but no significant difference was reported for near accommodative facility (myopes: 6.4 ± 1.8 cpm; emmetropes: 7.0 ± 1.5 cpm) (Pandian et al. 2006). Monocular accommodative facility testing using semiautomated flippers among 20 to 27-year-old adults before and after 20-minutes near task showed a significantly delayed negative response time by about 500 milliseconds in both emmetropes and myopes (p = 0.04), although myopes showed had a faster negative response time compared to emmetropes (p = 0.001). However, no significant differences existed in baseline monocular near accommodative facility and response times between the refractive groups before the near task (Jiang and White 1999). On comparing the monocular accommodative response times for distance viewing, O’Leary and Allen (2001) reported a significantly delayed positive response time in myopes (13.1 ± 21.2 seconds) when compared to emmetropes (6.49 ± 15.0 seconds, p < 0.05); both positive and negative response times were delayed in myopes when compared to emmetropes in other studies by about 1000 milliseconds (Pandian et al. 2006) and 500 milliseconds respectively (Radhakrishnan et al. 2007). None of these studies reported a significant difference in response times between the myopic and emmetropic groups for near accommodative facility testing. Allen and O’Leary (2006) examined the relationship between accommodative functions and myopia progression among 18 to 22-year-old young adults and showed that near monocular accommodative facility and binocular lag of accommodation were significant predictors of myopia progression.

Earlier studies show that distance accommodative facility differs significantly with refractive error in pre-adolescent children (6–8-year-olds) or young adults (18–35-year-olds). However, it is unclear whether monocular near accommodative facility is different between myopes and emmetropes during adolescence. Late-onset myopia that develops after 15 years of age is linked to environmental factors, namely excessive nearwork (McBrien and Millidot 1986). Accommodation is the key factor that links nearwork and myopia; reduced near monocular accommodative facility and higher lag of accommodation are significant predictors for myopic progression in young adults (Allen and O’Leary 2006). Thus, the present study sought to discover the relationship between near accommodative facility and myopia during adolescence. The aim and objective of this study is to measure and compare monocular near accommodative facility to find out the differences between myopic and emmetropic adolescents who are younger and older than 15 years of age.

## Methods

This was a cross-sectional, clinical study for which the participants were recruited from the outpatient clinic of Sankara Nethralaya, which mainly catered to the needs of patients with age-related cataracts and refractive errors. Children who came to the outpatient clinic only for primary eye care services, like routine eye examinations and annual refractive error monitoring, were invited to participate in the study. Participants with a low degree of myopia (≤ –0.50D and > –6.00D) as per the definition of the International Myopia Institute (Flitcroft et al. 2019) with a best-corrected visual acuity of 6/6 were included.

All participants underwent a baseline visual acuity assessment using a Bailey-Lovie log MAR chart and a preliminary ocular examination, including a slit lamp examination and a dilated fundus examination to exclude participants with any ocular pathologies. Binocular vision testing was carried out for all participants to exclude participants with any binocular vision anomalies.

Binocular vision testing included measuring the near point of accommodation and the amplitude of accommodation using a push-up test, pencil push-up test, and penlight red-green goggle test for a near point of convergence measurement, and a phoria and gradient AC/A ratio measurement using Maddox rod technique. Suppression was checked by asking participants if they perceived both red and green colored lights while performing the pen-light red-green goggle test and if they perceived the spotlight and the streak of light during the Maddox rod test.

Participants were classified into two different age groups based on the cut-off criteria recommended by the American Academy of Pediatrics (Hardin et al. 2017), namely: (i) Early adolescence (11–14 years), (ii) Middle adolescence (15–17 years), and (iii) Late adolescence (18–21 years). Our two groups were (i) Younger adolescents: Adolescents younger than 15 years (range: 11–14 years), and (ii) Older adolescents: Adolescents aged 15 years and older (range: 15–21 years). Middle and late adolescents were grouped together to set the cut-off age at 15 years for the older adolescent group. Participants were classified into myopic and emmetropic groups based on their cycloplegic spherical equivalent refraction: (i) Myopes (SE ≥ –0.50D to –6.00D) and (ii) Emmetropes (SE –0.25D to +0.75D).

### Refractive error measurement

Refractive error was measured objectively by retinoscopy (the Welch Allyn technique), followed by subjective refraction. Defogging was done to achieve a subjective end point, and it was verified using the duochrome test. All participants underwent a cycloplegic refraction as per the guidelines of the outpatient department for examining pediatric patients. Cycloplegia was achieved by instilling two drops of 1% cyclopentolate hydrochloride and one drop of 1% tropicamide, with the drops being administered in five minute intervals. Cycloplegic refraction was carried out after 30 minutes from instilling the last drop.

### Accommodative facility measurement

Monocular near accommodative facility was measured using flippers, with the best-corrected spectacle refraction for all participants. Monocular facility was measured in order to eliminate the effect of convergence during binocular facility testing. The non-tested eye was occluded. Monocular accommodative facility was measured for a duration of 60 seconds, using a +2.00D/–2.00D handheld flipper and a near point card with N6 print held at 40cm for all participants. The participants were instructed as follows:

“You should look at the smallest row of letters and try to keep them clear. Two types of lenses will be flipped in front of your eyes. Initially, the letters may remain blurred for few seconds. They will become clear after a few seconds. You should say, ‘clear’ as soon as the letters become clear through both lenses. The test will be done for one minute, and the number of flips will be counted”.

Participants were given a practice run for half a minute before the actual measurements were made.

The total number of cycles over a 60-second period was measured. When the subject was able to clear both lenses, the facility was counted as one full cycle. When the subject was able to clear only one side of the flipper — either the plus or minus side — facility was counted as a half cycle. When the subject was unable to clear the lens within the total 60 seconds, the facility was recorded as zero. The unit of accommodative facility was cycles per minute (cpm).

### Statistical analysis

Data on near accommodative facility was analyzed using SPSS Version 14.0. The association between the distribution of accommodative facility, refractive groups, and age groups was analyzed using Chi-square test of association. Data was analyzed to determine whether accommodative facility differed between age groups and refractive groups. Univariate Analysis of Variance was carried out with near accommodative facility as the dependent variable. Age and refractive error were the independent variables. Subgroup comparisons between age groups (younger adolescents and older adolescents) and between refractive groups (myopes and emmetropes) were done using independent samples t-tests. Post-hoc multiple comparisons for differences between age group and refractive groups — namely, younger adolescent myopes, older adolescent myopes, younger adolescent emmetropes, and older adolescent emmetropes were done using a one-way ANOVA with Bonferroni correction.

## Results

A total of 134 participants were recruited, out of which 15 were excluded due to other conditions listed in Table 1. 119 participants (myopes = 85; emmetropes = 34) aged 11–21 years participated in the study. Myopes had a mean age of 15.39 ± 2.71 years (age range: 11 years to 21 years) and a mean spherical equivalent refraction of –2.47 ± 1.71D (range: –0.50D to –4.50D) and –2.52 ± 1.71 D (range: –0.50D to –4.00 D) in the right and left eyes respectively. The mean age of emmetropic participants was 15.64 ± 3.29 years (age range: 12 years to 21 years), and the mean spherical equivalent refraction was 0.06 ± 0.19D (range: 0 D to +0.75D) and 0.03 ± 0.14 D (–0.25D to +0.75D) in the right and left eyes respectively.

**Table 1:** Exclusion criteria with number of subjects excluded.


EXCLUSION CRITERIA	NUMBER OF SUBJECTS EXCLUDED

Astigmatism greater than 3.00D	3

Myopia > –6.00D	2

Non Strabismic Binocular vision anomalies	6

Pseudo-myopia	3

Anisometropia	1

Amblyopia (Refractive, strabismic and mixed)	None

Keratoconus	None

Ocular pathologies	None


There were five myopes who could not clear the lenses within the one-minute test, of which two were in the younger adolescent group, and the remaining three were older adolescents. All emmetropes were able to clear the lenses within the minute.

**Figure 1:**
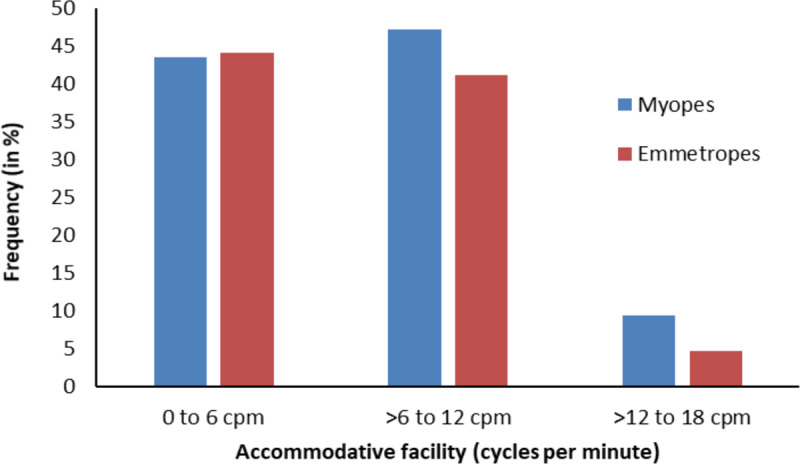
Frequency distribution for monocular near accommodative facility in myopes and emmetropes.

The frequency distribution difference of monocular near accommodative facility between myopes and emmetropes is shown in Figure 1. There was no significant association between the distribution of accommodative facility and refractive groups (χ^2^ = 0.81, df = 2, p = 0.67), with an accommodative facility of 0– 6 cpm being equally common in both myopes and emmetropes. The percentage of myopes and emmetropes with monocular near accommodative facility between 0–6 cpm was myopes: 43.5% and emmetropes 44.1%, greater than 6 and up to 12 cpm – myopes: 47.1% and emmetropes 41.2%, and a facility of more than 12 and up to 18 cpm – myopes: 9.4% and emmetropes 14.7%.

**Figure 2:**
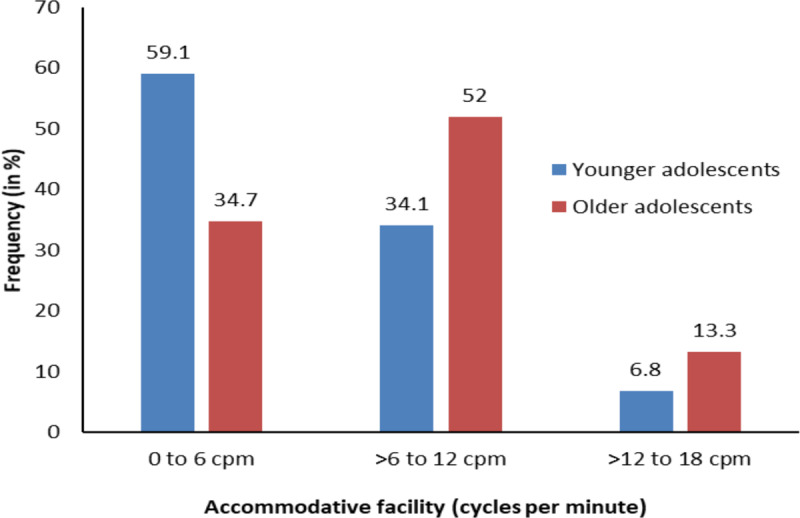
Frequency distribution for monocular near accommodative facility in younger adolescents and older adolescents.

The frequency distribution difference between the younger adolescent age group and the older adolescent age group for monocular near accommodative facility is shown in Figure 2. There was a significant association between the distribution of accommodative facility and age groups (χ^2^ = 6.82, df = 2, p = 0.03). 59.1% of younger adolescents and 34.7% of older adolescents had a monocular near accommodative facility between 0–6 cpm; 34.1% of younger adolescents and 52% of older adolescents had a facility of greater than 6 and up to 12 cpm, and only 6.8% of younger adolescents and 13.3% of older adolescents had a facility of more than 12 and up to 18 cpm. Accommodative facility of 0–6 cpm was more common in younger adolescents than older adolescents.

**Figure 3:**
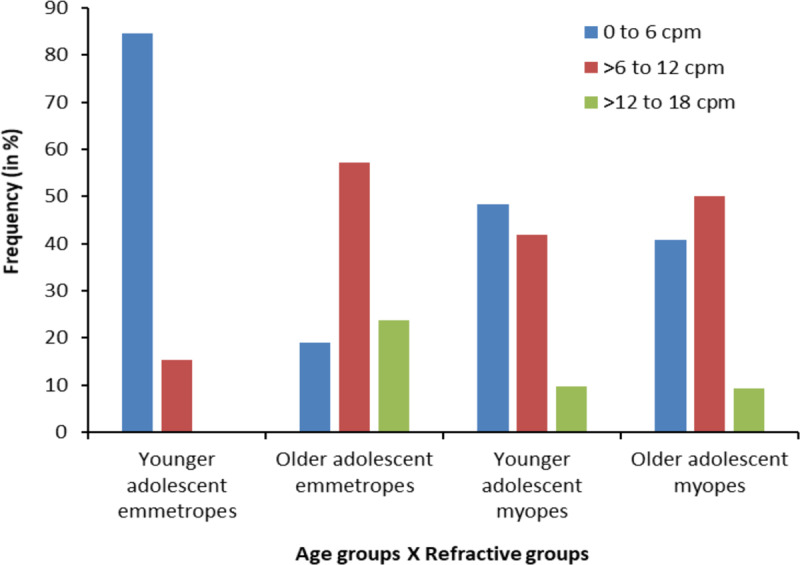
Frequency distribution for monocular near accommodative facility in younger adolescent and older adolescent emmetropes and myopes.

The frequency distribution for monocular near accommodative facility in younger and older adolescent myopic and emmetropic groups is shown in Figure 3. There was a significant association (χ^2^ = 15.74, df = 6, p = 0.015), with 84.6% of the younger adolescent emmetropes having 0–6 cpm, while 15.4% of them had greater than 6 and up to 12 cpm, and none of them had accommodative facility greater than 12 and up to 18 cpm. In the older adolescent emmetropic group, only 19% of the participants had accommodative facility of 0–6 cpm, while 57.2% of them had accommodative facility greater than 6 and up to 12 cpm, and 23.8% of them had greater than 12 and up to 18 cpm. In the younger adolescent myopic group, 48.4% had accommodative facility of 0–6 cpm, 41.9% had greater than 6 and up to 12 cpm, and 9.7% had more than 12 and up to 18 cpm. In the older adolescent myopic group, the percentage of participants with accommodative facility between 0– 6 cpm, greater than 6 and up to 12 cpm, and more than 12 and up to 18 cpm were 40.7%, 50%, and 9.3% respectively. Monocular near accommodative facility of 0–6 cpm was more common in the younger adolescent emmetropic group followed by the younger myopic adolescent group.

**Figure 4:**
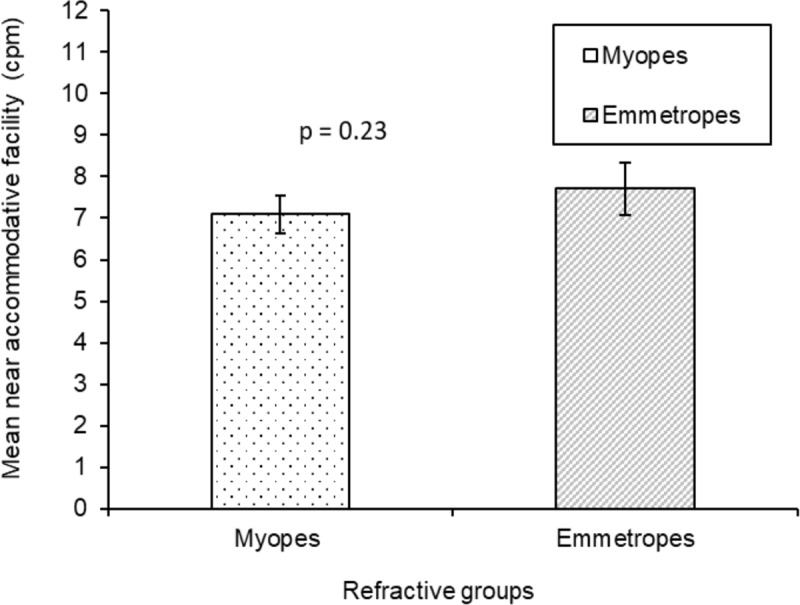
Comparison of mean monocular near accommodative facility between myopes and emmetropes (Error bars denote Standard Error).

**Figure 5:**
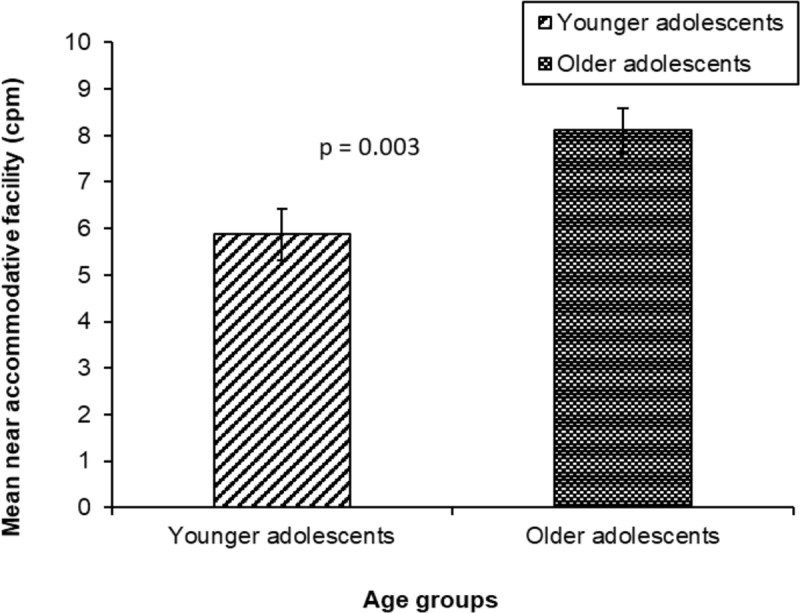
Comparison of mean monocular near accommodative facility between young and older adolescents (Error bars denote Standard Error).

The mean monocular near accommodative facility for the overall sample was 7.26 ± 4.10 cpm. The effect of refractive group on near accommodative facility was not significant (F_1,115_ = 0.067; P = 0.80), with no significant difference in mean monocular near accommodative facility between myopes (7.09 ± 4.23 cpm) and emmetropes (7.71 ± 3.67 cpm, p = 0.23). (Table 2 and Figure 4). Age group had a significant effect on near accommodative facility (F_1,115_ =13.44; P = 0.0001), with a significantly lower cpm in young adolescents (5.87 ± 3.72 cpm) compared to older adolescents (8.11 ± 4.11 cpm, p = 0.003) (Table 2 and Figure 5).

**Table 2:** Comparison of mean monocular near accommodative facility between different age groups and refractive groups.


	NEAR ACCOMMODATIVE FACILITY IN CPM (MEAN ± SD)	RANGE (IN CPM)

Myopes (n = 85)	7.09 ± 4.23	0 to 16.5

Emmetropes (n = 34)	7.71 ± 3.67	2 to 16.5

	P = 0.23	

Younger adolescents (age ≤ 14 years) (n = 44)	5.87 ± 3.72	0 to 16.5

Older adolescents (age ≥ 15 years (n = 75)	8.11 ± 4.11	0 to 16.5

	P = 0.003	

Younger adolescent myopes (n = 31)	6.48 ± 4.12	0 to 16.5

Older adolescent myopes (n = 54)	7.44 ± 4.35	0 to 16

Older adolescent emmetropes (n = 13)	4.77 ± 2.05	3.0 to 9.0

Older adolescent emmetropes (n = 21)	9.52 ± 3.27	4.5 to 15

	P = 0.005	


* cpm –cycles per minute; †SD – Standard deviation.

**Figure 6:**
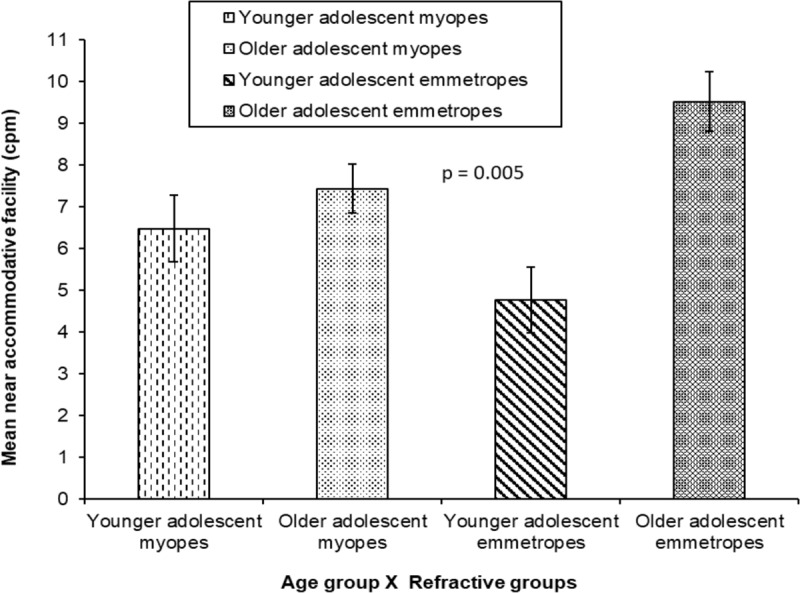
Comparison of mean monocular near accommodative facility between younger adolescents and older adolescent myopic and emmetropic groups (Error bars denote Standard Error).

There was a significant interaction ie., a combined effect of age group and refractive group on near accommodative facility (F_1,115_ = 4.60; P = 0.03) (Table 2 and Figure 6), with significant differences in mean monocular accommodative facility between younger adolescent emmetropes (4.77 ± 2.05 cpm) compared to older adolescent emmetropes (9.52 ± 3.27 cpm, p = 0.005). Similarly, younger adolescent myopes (6.48 ± 4.12 cpm) also had a significantly lower monocular near accommodative facility when compared to older adolescent emmetropes (9.52 ± 3.27 cpm, p = 0.02).

Monocular near accommodative facility was not significantly different between younger adolescent myopes (6.48 ± 4.12 cpm) when compared to older adolescent myopes (7.44 ± 4.35 cpm, p = 1.00). No significant difference in monocular near accommodative facility was found between older myopic adolescents (7.44 ± 4.35 cpm) and older emmetropic adolescents (9.52 ± 3.27 cpm, p = 0.25); younger emmetropic group (4.77 ± 2.05 cpm) did not differ significantly from older myopic adolescents (7.44 ± 4.35 cpm; p = 0.11).

## Discussion

The results of the present study show a significantly reduced monocular near accommodative facility in the younger adolescent emmetropic group compared to the older adolescent emmetropic group. But monocular near accommodative facility was not significantly reduced in the younger adolescent age group when compared to the older group the myopic group. This shows that age group has differential effects on monocular near accommodative facility within the emmetropic group but not the myopic group.

The younger adolescent myopic group showed a significantly reduced monocular near accommodative facility when compared to the older adolescent emmetropic group, but not when compared to the older adolescent myopic group. These findings show that age group and refractive group together can impact monocular near accommodative facility, thus showing some evidence for the effects of interaction between age and refractive error on monocular near accommodative facility. Pandian et al. (2006) did not find any interaction between age and refractive error in their study group, possibly because their study included 6–8-year-old children and did not include adolescents. In the entirety of their younger cohort, there were only 20 myopes (1.5%) while about 74% were emmetropic. On the contrary, in the current study, 71% were myopes. Thus, the reported difference could be due to differences in the distribution of refractive errors between children and adolescents.

In this study, monocular near accommodative facility was not different between the myopic and emmetropic groups. This is consistent with previous studies, which also showed no refractive group difference (Pandian et al. 2006; O’Leary and Allen 2001; Radhakrishnan, Allen and O’Leary 2007). The present study has shown a reduced accommodative facility in younger adolescents compared to older adolescents, a finding similar to that by Pandian et al. (2006), who showed an increase in monocular near accommodative facility with increasing age. Increased accommodative facility in the older adolescent group could be because accommodation stabilizes with age, with children younger than 12 years of age showing reduced monocular near accommodative facility compared to adults (Scheiman et al. 1988; Jimenez et al. 2003).

It is worthy to note that myopes and emmetropes showed no significant refractive group difference. However, when subdividing the groups further into different age groups, 11–14-year-old younger adolescent emmetropes showed a significant difference compared to 15–21 year-old older adolescent emmetropes. On the other hand, within the myopic group, no significant difference was obtained between young adolescents myopes compared to older adolescent myopes.

Previous studies on accommodative facility testing have reported that differences in target size, target distance, test lens power, variations in test duration and time of testing affect accommodative facility (Rouse et al. 1989; Siderov and Johnston 1990; Rouse et al. 1992; Wick et al. 2002). A low-powered test lens, closer testing distance, and a larger target size will increase the number of cycles (Siderov and Johnston 1990; Rouse et al. 1992). Previous research also shows that both monocular and binocular accommodative facility increases with extended test duration (Rouse et al. 1989; Rouse et al. 1992). In the present study, the target size, test distance, test lens power, and the test duration were kept constant for all participants; hence it is unlikely that the results of the present study are influenced by changes in these parameters.

In the present study, the mean near accommodative facility was 7.09 cpm for myopes and 7.71 cpm for emmetropes, which is lower than the values reported by O’Leary and Allen (2001) for myopic (11.4 cpm) and emmetropic (12.9 cpm) young adults aged 18–27 years. The mean monocular near accommodative facility of young adolescents aged 11–14 years old in the current study was 5.87 cpm, which is lower than the mean monocular facility rate at near reported by Pandian et al. (2006) for the 7-year-olds (mean 7 cpm) and the 8-year-olds (mean 7.6 cpm). Previous studies have used N5 target (Pandian et al. 2006; O’Leary and Allen 2001) and measured facility at 33 cms (Pandian et al. 2006). In the current study, although a larger target size (N6) should have increased the accommodative facility, this was less likely to be the case, since a farther testing distance (40 cm) would have offset this effect.

The mean facility rate for the overall sample in the current study is lower than the previously reported monocular near accommodative facility values of 11 and 14 cpm among 7–12-year-olds and 13–17-year-old South Indian children respectively (Hussaindeen et al. 2017). This could be because the previous study used larger target sizes of 20/40 and 20/30 respectively for the younger and older cohorts. A larger target size may have resulted in increased cpm. Our findings are also lower compared to that reported among 8 to 12-year-old cohorts (7 and 7.4 cpm) by Jimenez et al. (2003) and Scheiman et al. (1988) respectively. Based on our study results, we would expect the monocular near accommodative facility reported in these studies (Pandian et al. 2006, Jimenez et al. 2003; Scheiman et al. 1988) to be lower than our findings, since they had a younger cohort. The mean monocular near accommodative facility is reported to be 13.7 cycles per minute among 10–18-year-old subjects (Rouse et al. 1991). Despite having an older cohort aged 11–21 years, the mean facility rate for the overall sample in the current study is lower than that reported by Rouse et al. (1991). Jackson and Goss (1991) reported a monocular facility rate of 4.7 and 5.7 cycles (right and left eyes respectively) in half a minute among 8–16-year-olds. Considering that they tested only for half a minute, we would expect their findings to be higher than that reported in our study, assuming a test duration of one minute.

In the present study, although response times were not quantified, clinical reports suggest that younger adolescent myopes and emmetropes with reduced accommodative facility had difficulty clearing the positive lens side of the flipper with delayed negative response times. Jiang and White (1999) showed delayed negative response times in both myopic and emmetropic young adults for near viewing, although it was relatively faster in myopes; Pandian et al. (2006) and Radhakrishnan et al. (2007) showed delayed negative response times for distance viewing. Present study results show some evidence that the monocular negative response time may be delayed for near viewing in these adolescents. This may be explained by changes in proximal accommodation during near accommodative facility testing. Near target increases the proximal accommodation, whilst the imposed positive lens relaxes the accommodation thus making it difficult to relax the accommodation with these opposing cues, thereby leading to lengthy cycles. However, this needs to be investigated.

The results of this study add to the present knowledge about accommodative facility: monocular near accommodative facility is reduced in younger adolescent emmetropes when compared to older adolescent emmetropes. But such difference did not exist in the myopic group. However, the younger adolescent myopes did show a difference with reduced accommodative facility when compared to older adolescent emmetropes. This has not been reported previously.

These findings highlight the importance of near accommodative facility testing in clinical practice by optometrists while evaluating emmetropic and myopic adolescents aged 14 years and younger, since they show a reduced monocular near accommodative facility, which is reportedly a significant predictor for myopia progression. The study findings also stress the need for near accommodative facility testing — particularly its inclusion in the battery of accommodation tests in the myopia evaluation clinical protocol, along with accommodative lag when examining children and adolescents of different age groups. It is a simple and easy test that can be performed even in low- and medium-resource clinical settings.

There are however, several limitations of this study. Whilst the overall sample is large, there are smaller sample sizes in some groups, and the data is cross-sectional, so no longitudinal predictions can be made. It is possible that a smaller number of participants in the younger emmetropic group might have favored the results, and therefore, this needs to be examined in a larger number of participants. We could have measured response time to substantiate delayed negative response time, which can be done in future studies. Distance accommodative facility was not measured in this study, as this has been done before. Moreover, since near accommodative facility is linked to myopia progression in young adults (Allen and O’Leary 2006), the present study focused on differences in near accommodative facility for different refractive and age groups.

## Conclusion

To conclude, for this clinical sample, there was significantly reduced monocular near accommodative facility between younger adolescents and older adolescents. Emmetropes and myopes in the younger adolescent age group had reduced monocular near accommodative facility when compared to emmetropes in older adolescent age group, but not when compared to older adolescent myopes.
